# Are artificial agents perceived similarly to humans? Knowns, unknowns and the road ahead

**DOI:** 10.3389/fpsyg.2025.1565170

**Published:** 2025-08-07

**Authors:** Marius Rubo, Isabel Neumann

**Affiliations:** ^1^Cognitive Psychology, Perception and Research Methods, Institute of Psychology, University of Bern, Bern, Switzerland; ^2^Department of Clinical Psychology, and Psychotherapy, Institute of Psychology, University of Würzburg, Würzburg, Germany

**Keywords:** artificial agents, avatars, artificial intelligence, deception, demand characteristics

## Abstract

Starting long before modern generative artificial intelligence (AI) tools became available, technological advances in constructing artificial agents spawned investigations into the extent to which interactions with such non-human counterparts bear resemblance to human-human-interactions. Although artificial agents are typically not ascribed a mind of their own in the same sense as humans, several researchers concluded that social presence with or social influence from artificial agents can resemble that seen in interactions with humans in important ways. Here we critically review claims about a comparability between human-agent interactions and human-human-interactions, outlining methodological approaches and challenges which predate the AI era but continue to influence work in the field. By connecting novel work on AI tools with broader research in the field we aim to provide orientation and background knowledge to researchers as they move forward in inquiring how artificial agents are used and perceived, and to further contribute to an ongoing discussion around appropriate experimental setups and measures. We argue that both when confronting participants with simple artificial agents or AI-driven bots, researchers should (1) scrutinize the specificity of measures which may indicate social as well as more general, non-social processes, (2) avoid deceptive cover stories which entail their own complications to data interpretation and (3) see value in understanding specific social-cognitive processes in interactions with artificial agents even when the most generalizable comparisons with human-human interactions may not be achieved in a specific experimental setup.

## Introduction

When playing the game *20 questions*, what information would suffice to conclude that the correct answer must be “a human being”? Clearly, positive responses to the questions “Is it bigger than a book?” and “Is it warm?” would not suffice. Likewise, after hearing that the looked-for entity “can increase the heart rate in nearby people” and “may cause frustration when being non-responsive,” one would likely not yet be ready to make a definite guess but would instead consider further information to inquire. More specific (albeit not necessarily unique) characteristics of humans to inquire are a capability for experience (the capacity to *feel and sense*, e.g., to experience pain, feel calm, or to be conscious; Gray et al., 2007; Malle, 2021; Weisman et al., 2017) or a fundamental need to form bonds with others (Baumeister and Leary, 2017).

A topic which has long captured the attention of philosophers and anthropologists, the characterization of human beings received a new perspective through the advancement of artificial humanoid agents (Schneider, 2019). In addition to debates on when artificial agents should be seen as equivalent to humans, it was now also inquired to what extent such agents are perceived by people as if they were human. Researchers can quickly point to a profound distinction: regardless of whether artificial agents are displayed as characters on a computer screen or represented as robots (Oh et al., 2018; Pan and Hamilton, 2018), they may be ascribed a capacity to act and do (i.e., to make choices, recognize others, or remember things; Gray et al., 2007; Malle, 2021; Weisman et al., 2017), but are typically not ascribed a capability for an own *experience*. In fact, people were even found to express unease at the idea of ascribing experience to artificial agents (Gray and Wegner, 2012, MacDorman, 2024).

Despite this striking discrepancy in the perception of humans and artificial agents, researchers observed that people can have a sense of “being with another” or *social presence* when being co-located with an artificial agent (Bailenson et al., 2005; Biocca et al., 2001). A wide range of other similarities between human-agent-interactions and human-human interactions were reported in a large number of studies, spanning across different fields and covered in separate review articles (e.g., Chugunova and Sele, 2022; Felnhofer et al., 2023; Capraro et al., 2024). For instance, Nass et al. (1999) observed an adherence to social norms in participants who interacted with computer-controlled agents; Eyssel and Hegel (2012) found participants to apply gender stereotypes to virtual agents; Slater et al. (2013) found participants to attempt to stop an attack by one virtual agent on another; Iachini et al. (2016) and Zibrek et al. (2020) observed similar inter-personal distances to artificial agents in virtual reality (VR) as one would expected toward real people; Gonzalez-Franco et al. (2018) found participants to help an artificial agent in avoiding electric shocks; Wienrich et al. (2018) observed a social inhibition of return effect toward artificial agents; Broadbent (2017) observed a willingness to disclose emotional experiences toward an artificial agent; Karhiy et al. (2024) reported comparable levels of perceived stress after a mindfulness intervention between a teletherapist and virtual human; Rubo and Gamer (2020) found a reciprocation of eye-contact with an artificial agent when immersed in VR and Krämer et al. (2013) observed a reciprocation of an agent’s smiling behavior; Dechant et al. (2017) and Rubo and Munsch (2023) found a relative aversion of eye-contact with an artificial agent in people higher on social anxiety. In addition, several studies reported physiological (Kiilavuori et al., 2021; Pan et al., 2012; Wieser et al., 2010) and neural (Caruana et al., 2015; Kompatsiari et al., 2021; von der Pütten et al., 2014; Schilbach et al., 2010) reactions to interacting with artificial agents which resembled reactions to interacting with humans.

A large number of studies also observed differences in responses to artificial agents compared to humans. For example, participants behaved more selfishly in human-machine interactions compared to human-human interactions (e.g., Cohn et al., 2022; Chugunova and Sele, 2022); socially anxious individuals in a study by Kang and Gratch (2010) revealed more intimate information about themselves when interacting with a virtual character compared to when interacting with a real human, possibly reflecting a reduced fear of evaluation in interactions with mindless agents. If information was provided by a human-controlled compared to a computer-controlled agent, participants showed better results in a post-study test and higher levels of physiological arousal (Okita et al., 2007), and acted differently when protecting team partners in a cooperative game (Merritt and McGee, 2012). Participants also exhibited stronger physiological arousal when interacting with human-controlled compared to computer-controlled agents (Lim and Reeves, 2010; Ravaja et al., 2006), stronger electrodermal reactions to eye contact with a person compared to with a human-controlled virtual character (Syrjämäki et al., 2020) and stronger (left occipitotemporal) N170 responses in response to gaze from a supposedly human-controlled compared to a computer-controlled agent (Caruana et al., 2016).

Notwithstanding observations of differences in reactions to artificial agents and humans, it was proposed that artificial agents may typically be perceived as human-like when they contingently respond to one’s actions and fulfill expectations that one has toward other humans (Skarbez et al., 2017; Slater, 2009). Even more generally, the *Computers are Social Actors* (CASA) framework poses that humans tend to react similarly to artificial agents as to other humans, and that processing of social cues occurs automatically and mindlessly both when a conspecific is a real human or an artificial agent (Nass and Moon, 2000; Nielsen et al., 2022; von der Pütten et al., 2010; Xu et al., 2022). Note that similar models were proposed in the domain of human-human interactions which are thought to often follow mindlessly applied heuristics (Social Heuristics Hypothesis, Rand et al., 2014, Capraro, 2024). A more nuanced view, the Threshold Model of Social Influence (Blascovich, 2002) postulates that human-controlled virtual characters (termed avatars) exert a higher social influence on humans than computer-controlled agents due to their higher degree of perceived agency (here defined as extent to which virtual characters are perceived as a vivid person).

Recent meta-analyses observed an overall heightened self-reported sense of social presence toward avatars compared to agents but no differences in behavioral responses (Fox et al., 2015; Felnhofer et al., 2023), seemingly allowing for an integration of both models. The aim of the present commentary, however, is not to summarize data with regards to their fit to proposed models. Instead, we outline general conceptual and methodological challenges in the field which underlie a wide range of studies and which, we will argue, require careful consideration when interpreting individual studies as well as meta-analytic summaries.

## Research practices and methodological challenges

Several of the above-mentioned studies may raise the question as to what extent observed reactions are informative in evaluating a more general comparability of human-agent interactions with human-human interactions. In particular, one may object that recorded reactions in participants often captured some of the more mundane and fleeting aspects of social interactions such as politeness and mindless applications of gender stereotypes (e.g., Nass et al., 1994) but may have missed to address their more profound and meaningful levels. One may argue that stronger support for the CASA framework could come from observations of higher-level social-cognitive functions, such as an activation of social-evaluative processes, in interactions with virtual agents. Two particularly relevant lines of study here are experiments using the Cyberball paradigm (Hartgerink et al., 2015; Williams and Jarvis, 2006)—where participants are socially excluded in ball-tossing game—and the Trier Social Stress (TSST) (Allen et al., 2017; Kirschbaum et al., 1993)—where participants carry out a performance task in front of a committee (Fallon et al., 2016; Helminen et al., 2021). Both the Cyberball paradigm (Hartgerink et al., 2015; Jauch et al., 2022; Kothgassner et al., 2014; Zadro et al., 2004) and the TSST were carried out with virtual characters as conspecifics or as committee (Fallon et al., 2016; Helminen et al., 2021) and were reported to induce subjective and physiological stress responses even when participants were informed that agents were computer-controlled.

However, two types of methodological challenges may instill hesitation toward concluding that artificial agents can indeed trigger social-cognitive processes in a comparable manner as humans. Firstly, there is no agreement on the measures that should be used to assess social-cognitive processes (Sterna and Zibrek, 2021) and several frequently used measures may capture more general constructs. For instance, reactivity in heart rate or salivary cortisol levels (which are commonly observed in response to the TSST; Helminen et al., 2021) are not specific to social stress but can similarly be seen in response to non-social tasks (Bozovic et al., 2013; Taelman et al., 2010). Responses to questionnaires which are intended to represent social cognition are likewise not immune to capturing more general constructs. For instance, Cyberball studies commonly use as a main outcome response a scale which is designed to assess threats to fundamental human needs but may more generally capture a sense of aversion (toward being unable to participate in ball-throwing while having no other task; Gerber et al., 2017). Several items may more generally infer frustration (e.g., “I felt somewhat frustrated during the Cyberball game”). Rubo and Munsch (2023) observed that the meta-analytically described effects of the Cyberball paradigm (Hartgerink et al., 2015) are cut in half when excluding this scale. Other measures which are assessed in response to the Cyberball task may similarly not necessarily reflect social-cognitive processes toward artificial agents. For instance, when asking about feelings of anger, responses may again reflect frustration with the testing situation rather than with emotions toward the virtual agents per se.

Secondly, even when assessing variables which more clearly reflect social-cognitive processes toward others, it can become intricate to assess the extent to which they are caused by a virtual agent rather than any other persons involved in the testing situation. Note that experimenters may not only influence participants’ behavior when they are visible, but also when they are present in the room but invisible to participants (Gallup et al., 2019) or even when their presence is merely implied, e.g., when participants are aware that a recording of their behavior may be viewed at a later point (Garcia-Leal et al., 2005; Gobel et al., 2015; Jones et al., 1997). When comparing interactions with artificial agents to interactions with humans, researchers therefore often strive to remove such effects by exposing all participants to the same form of interaction—which typically involves no contact with another person—and creating experimental manipulations merely by means of varying instructions given to participants. Specifically, participants in a range of experiments interacted with computer-controlled artificial agents after either receiving veridical information about the situation or after being erroneously informed that the agents were controlled by humans (Hartgerink et al., 2015; Jauch et al., 2022; Kothgassner et al., 2017; Syrjämäki et al., 2020). Such deceptive instructions were used in 19 out of 20 studies included in a recent meta-analysis on social responses toward computer-controlled agents and (supposed) human-controlled avatars (Felnhofer et al., 2023). Deceptive or misleading cover stories are sometimes complemented with additional steps to increase their credibility and reduce questioning by participants. For example, participants may be presented with supposed confederates controlling the avatar (Okita et al., 2007), experimenters leaving the room to check up with the confederate supposedly controlling the virtual character (Caruana et al., 2017), showing a supposedly live footage of the confederate (Neumann et al., 2023), waiting for another participant to control the virtual character (Weibel et al., 2008), and instructions providing explanations on how the other person could control the avatar (Caruana et al., 2017; Lucas et al., 2019).

Note how interpretations of research outcomes can hinge on participants’ belief in such cover stories. While comparable behavior toward agents and avatars (Fox et al., 2015; Felnhofer et al., 2023) can be interpreted to align with CASA if assuming that participants trust the cover story (indicating that social behavior is largely automatic and applied mindlessly even toward artificial agents), participants’ suspicion in the deception (Davidson et al., 2019)—which is only applied in one of the two conditions—may effectively eliminate the difference between the two conditions when participants are similarly aware of their interacting with artificial agents in both conditions. More so, deception can introduce its own measurement biases which can vary between participants (Hertwig and Ortmann, 2008; Kelman, 2017). While some participants who detect deceit may correspondingly act toward artificial agents as being mindless entities, others may nonetheless act according to what they believe is expected from them. The presence of such demand characteristics (Nichols and Maner, 2008; Lush, 2020) may more strongly influence explicit responses—thus explaining why self-reported social presence toward avatars was often higher compared to toward agents (Fox et al., 2015; Felnhofer et al., 2023)—although they can also affect more implicit measures (Vecchione et al., 2016). In addition, some participants may experience negative emotional reactions to detecting deceit (Walczyk and Newman, 2020).

It is often difficult to assess the proportion of participants who harbored suspicion toward a cover story since the use of standardized manipulation checks are rare in the field. Some studies do not inquire belief in the manipulation (e.g., Appel et al., 2012; Kothgassner et al., 2014; Lim and Reeves, 2010; Lucas et al., 2019), while other studies inquire participants’ understanding of the proposed situation but do not directly test belief in the cover story (Kothgassner et al., 2019). Even when the manipulation succeeds in eliciting statistically significant differences in how the interaction with the virtual character is perceived, belief in the manipulation may only be moderately strong (e.g., Felnhofer et al., 2018). Only in few studies were participants proactively approached by experimenters if they seemed suspicious about the cover story (Jauch et al., 2022; Neumann et al., 2023; Weibel et al., 2008).

Note that methodological problems which arise from falsely introducing computer-controlled agents as human-controlled avatars can similarly appear when falsely introducing human-controlled avatars as computer-controlled agents. In the case of TSST setups which are carried out with artificial agents as committee, researchers again removed all other humans from the testing room and informed participants that they were interacting with artificial agents in solitude. In reality, experimenters were still listening to participants while hiding in another room or behind a one-way mirror (Helminen et al., 2021), secretly controlling the agent’s behavior in what is referred to as a wizard of oz. setup (Pan and Hamilton, 2018). Here again, attributions of social stress in participants to their interactions with artificial agents hinges on their belief in an erroneous cover story.

## Considerations for future research

In the light of methodological challenges, we propose guidelines for future comparisons between interactions with computer-controlled artificial agents and humans or human-controlled avatars. Firstly, as argued in other fields within the behavioral sciences, deceptive cover stories should be avoided when possible as they can impair experimental control and undermine participants’ trust in psychological experimentation in the long run (Hertwig and Ortmann, 2008; Kelman, 2017). Researchers may argue that some questions cannot be addressed without the use of deception (Weiss, 2001). For instance, if the goal is to compare reactions to artificial agents and humans while holding all physical stimuli equal, one may quickly find oneself designing experiments where participants in all conditions are confronted with computer-controlled agents and receive diverging instructions about the character of the interaction in different conditions. While such setups may continue to have their place in the field—and may more explicitly explore and attenuate confounding effects resulting from deception—it may be noteworthy that alternatives exist. Specifically, research with no use of deception (i.e., comparing interactions with humans who are truthfully introduced as humans to interactions with artificial agents which are likewise truthfully introduced as what they are) need not be seen as methodologically inferior. Several authors have comprehensively outlined how such investigations, which assess unadulterated cognitive processes as they occur naturally in specific contexts, can be particularly informative for constructing cognitive theories (Beller et al., 2012; Hutchins, 2010; Kingstone et al., 2008; Miller et al., 2019). For such comparisons, researchers might more strongly incorporate observations from outside of the laboratory—such as when (anonymously) logging interactions with service robots or agents in computer simulations in comparison to similarly structured interactions with humans—in order to mitigate influences of demand characteristics and other experimenter effects (Nichols and Maner, 2008).

While interactions among humans are often characterized by their repeating occurrence (e.g., in the family or among colleagues), and problematic real-life phenomena such as ostracism likewise tend to span over larger time periods (Riva et al., 2016), research comparing effects of interacting with humans and artificial agents more commonly assessed relatively short-term consequences (e.g., mood, behavior, and physiological reactions during and within an hour after an experiment; Felnhofer et al., 2023; Fox et al., 2015). Future research may profit from more strongly incorporating a longer-term perspective when assessing the extent to which artificial agents are perceived similarly as humans. In particular, to test the hypothesis of a more general equalization of the perception of artificial agents with humans, researchers could test if people can form attachments to artificial agents similarly as to other humans (Levy et al., 2010) or if people can satisfy their need for longer-term bonds (Baumeister and Leary, 2017) in interactions with artificial agents. Considering that building a long-term relationship takes time for bonds and trust to develop, key factors should be considered regarding adapting the behavior of the virtual character to the current closeness and status of the relationship, such as memory of previous encounters (Kasap and Magnenat-Thalmann, 2012), but also social attitudes (Ben Youssef et al., 2015) and personal self-disclosure (Wu et al., 2024).

Researchers who aim to assess the comparability of interactions with humans and artificial agents in the most general sense (e.g., assessing the general magnitude of social influence from them; Fox et al., 2015; Felnhofer et al., 2023) will continue to be tasked with defining a set of outcome variables which is needed for such general considerations. Similarly as in the 20 questions game, skeptics may remain doubtful if the acquired information suffices to justify a definite guess, or if instead others outcome variables may still need to be considered. Note that the metaphor of the 20 question game was used in a discussion on whether researchers should even see themselves as engaging in such a game when carrying out research (i.e., aiming at a definite model which unifies all observations), or whether it may be more fruitful to accept certain levels of multiplicity when unification is out of sight (Newell, 1973). Similarly, other authors have since highlighted the value of understanding individual phenomena as they occur in specific situations, encouraging researchers to refrain from obstinately aiming for unified theories (Shapiro, 2009; Skarbez et al., 2021). Researchers may likewise investigate a variety of individual questions such as the utility of artificial agents in social skills training (Howard and Gutworth, 2020) and the service industry (Pelau et al., 2021) or the characteristics which make artificial agents desirable to interact with (Hildt, 2021)—without directly aiming at testing a more general equitability of artificial agents with humans.

## Moving forward in the AI era

While interactions with artificial agents were investigated since the 1960s (Agassi and Wiezenbaum, 1976), advances in generative AI, in particular large language models (LLMs) such as ChatGPT which allow for rich text-based interactions, stimulated a new and ongoing wave of research in a wide range of fields (Carlbring et al., 2023; Rebelo et al., 2023; Hudecek et al., 2024). For instance, AI tools are expected to augment or monitor processes in health care while allowing professionals to spend more time with patients, effectively alleviating personnel undersupply (Augurzky and Kolodziej, 2018; Rabbitt et al., 2015). Some studies in the field focused on the usefulness of such chatbots as an interactive text-based knowledge resource (Wester et al., 2024), but several studies also tapped into the question of how human-like AI chatbots are perceived. It was observed that AI chatbots may be rated as relatively human-like in their role as therapist during counseling sessions (Vowels et al., 2024), but interacting with them may also require an acclimatization period for users (Araujo and Bol, 2024). In addition, a chatbot’s expressions of empathy may be perceived as inauthentic (Seitz, 2024) and people do not trust AI chatbots in the same sense as other humans (Montag et al., 2024).

Although the introduction of AI technology to artificial agents constitutes a groundbreaking advancement in the field, investigations into perceptions of and reactions to such chatbots may profit from research practices and experiences gained in research predating the AI era. In particular, while interactions with AI chatbots are rarely contrasted with human-human interactions in direct juxtaposition, past investigations into perceptions of artificial agents (with no AI capabilities) often incorporated experimental variations to allow for the most direct comparison between human-agent and human-human interactions (Felnhofer et al., 2023). Research on interactions with AI chatbots may incorporate similar experimental setups in order to collect informative data on the perceived humanness of such agents.

Since interactions with modern AI can closely resemble human-human interactions in their content but may also confront participants with misinformation beyond experimenters’ control, it was argued on ethical grounds that participants should be consistently made aware when they interact with AI agents as opposed to human counterparts (Piñeiro-Martín et al., 2023; Tabassum et al., 2025)—a guideline which prohibits the implementation of deceptive cover stories. Note, however, that experimental researchers using AI tools may more easily abandon the practice of misleading participants into thinking that a computer-driven agent was human-controlled: while this technique sometimes appeared necessary to provoke reactions in participants when computer-controlled agents lacked capabilities to display human-like behavior, AI chatbots may exhibit sufficient interactional realism to elicit complex social reactions even when transparently introduced to participants as artificial agents. In addition to previous research, reactions to AI chatbots may then be tested on a wider range of measures rooted in psychological theory including higher-level social processes such as reactions to social evaluation (Allen et al., 2017) or the need to be sensed and attended to by others (Baumeister and Leary, 2017) as well as physiological (Syrjämäki et al., 2020) and neural (Caruana et al., 2016) reactions to social encounters.

Importantly, researchers investigating reactions to AI chatbots may take up and continue the discussions around criteria by which the perceived humanness of AI agents should be evaluated (Rubo and Munsch, 2023). Modern technology may furthermore help to avoid experimenter effects which may have influenced previous laboratory research on reactions to artificial agents (Gallup et al., 2019) since AI tools can now be more easily disseminated to participants’ smartphones and tested in everyday situations with less experimenter influence. While AI will continue to enrich and stimulate a range of research fields, it can also allow for more rigorous basic research into human social processes by profiting from and extending on past research conducted before the AI era.

## Conclusion

The goal to compare people’s perception of artificial agents with the perception of other humans has inspired a considerable amount of research and unearthed a range of interesting findings, several of which were taken to suggest a comparability of the two. The field was also confronted with major challenges: firstly, it proved difficult to specify what type of results would imply a more general comparability between the perception of humans and artificial agents. Secondly, it remained difficult to thoroughly remove contamination of experimenter and demand characteristics effects from observations. We suggest that future research may avoid the use of deceptive cover stories and the presence of experimenters by investigating interactions in more natural environments outside of laboratory setups. Assessed outcomes may more strongly extend toward longer-term phenomena such as the development of interpersonal bonds. We argue that researchers need not necessarily strive for the most global comparisons but may focus on understanding individual facets of interactions between humans and artificial agents. Moving to a new era in the field where artificial agents can be endowed with the capability for naturalistic participation in interactions using AI models, researchers can significantly enhance our understanding of social-cognitive processes in interactions with artificial agents, both drawing on and extending research practices from more traditional work in the field.

## Data Availability

The original contributions presented in the study are included in the article/supplementary material, further inquiries can be directed to the corresponding author.
